# Added Value of Biological Effective Dose in Dosiomics-Based Modelling of Late Rectal Bleeding in Prostate Cancer

**DOI:** 10.3390/cancers16244208

**Published:** 2024-12-17

**Authors:** Christian A. M. Jongen, Wilma D. Heemsbergen, Luca Incrocci, Ben J. M. Heijmen, Linda Rossi

**Affiliations:** Department of Radiotherapy, Erasmus MC Cancer Institute, University Medical Center Rotterdam, Dr. Molewaterplein 40, 3015 GD Rotterdam, The Netherlands

**Keywords:** dosiomics, texture analysis, late rectal bleeding, radiobiological modelling, NTCP

## Abstract

Background/Objectives: Extracting spatial features (texture analysis) from dose distributions (dosiomics) for outcome prediction is a rapidly evolving field in radiotherapy. To account for fraction size differences, the biological effective dose (BED) is often calculated. We evaluated the impact and added value of the BED in the dosiomics prediction modelling of grade ≥ 2 late rectal bleeding (LRB) probability within 5 years after treatment in three parts. Methods: For N = 656 prostate cancer patients previously treated in a randomized trial with conventional (CF) or hypofractionated (HF) radiotherapy, 42 dosiomic features were extracted from the dose distributions of the delineated rectum in physical doses and from dose distributions converted to the BED. Part 1: To assess whether an HF BED dosiomics model is generalizable to CF and vice versa, multivariate logistic regression BED models were constructed for HF and CF separately and tested on the other fractionation scheme. Part 2: The BED models were fitted to combined HF and CF data together to test whether this resulted in better models. Part 3: Separate physical HF and CF models were constructed and compared to the BED models. Results: Part 1: Dosiomics related to large-zone and long-run high-dose levels were predictive for both HF and CF. Deviation from the mean gray level was only predictive for HF. The BED HF model calibrations with CF data and vice versa were generally poor. AUCs ranged from 0.55 to 0.65. Part 2: Compared to the separate models, the models fitted to the combined HF and CF data showed better discriminative ability in CF but not in HF. Part 3: The apparent performances of models for the BED and physical dose were similar. Conclusions: Using the BED in the predictive dosiomic modelling of late rectal bleeding after prostate cancer radiotherapy to account for differences in fraction doses was of limited value.

## Simple Summary

By identifying patterns and voxel correlations (texture analysis), it is possible to characterize dose distributions. These dose parameters are called dosiomic features. The linear-quadratic model is a widely applied model in radiotherapy to calculate the biological effective dose for a tissue of interest based on the fraction size sensitivity, fraction size and total dose. In this study, we investigated the added value and impact of applying biological effective dose to dosiomic features in prediction models.

## 1. Introduction

Texture analysis is a method in which textural features of an image are computed and used to characterize the image [1]. The technique has been applied across several medical imaging techniques and organs for purposes such as segmentation, discrimination between healthy and diseased tissue and the characterization of tumors and pathologies [2]. Texture analysis is also applied to 3D dose distributions of normal tissues to obtain so called ‘dosiomic’ features for outcome prediction modelling [3,4,5]. Dosiomic features capture dose voxel patterns and contain spatial information of the dose distribution, contrary to traditional dose parameters.

Rossi et al. developed normal tissue complication probability (NTCP) models for genitourinary and gastrointestinal endpoints and showed that dosiomics can add value to models based on traditional dose parameters [3]. Also, for the radiation pneumonitis endpoint, Sheen et al. demonstrated that hybrid models show better predictive power compared to models based on clinical and conventional dose parameters only [6].

With the large-scale clinical introduction of hypofractionation in radiotherapy with multiple applied fractionation schemes for various tumor sites, there is a drive and opportunity for developing prediction models that work for all schemes. The linear–quadratic model (LQ-model) is a widely applied model in radiotherapy to calculate biological effective dose (BED) for a tissue of interest based on the fraction size sensitivity (α/β), fraction size and total dose. There are several dosiomics-based NTCP models available in the literature that are based on doses that are corrected for fractionation using the LQ-model [7,8,9]. However, to the best of our knowledge, there are no studies in which dosiomics-based models have been developed using data of one fractionation scheme and tested on another.

In this study, the impact and added value of the linear–quadratic model in dosiomics-based late rectal bleeding (LRB) NTCP modelling was investigated in three parts, using data from a previous randomized clinical trial. Part 1: to study whether dosiomics-based models are generalizable to other fractionation schemes, BED models developed with hypofractionation (HF) data were tested with conventional fractionation (CF) data and vice versa. Part 2: to assess the value of combining datasets, the BED models were developed with combined HF and CF data and tested with HF and CF separately. Part 3: separate physical dose models were developed for HF and CF and compared to the BED models.

## 2. Materials and Methods

### 2.1. Patients

The data used for modelling consisted of 656 intermediate- to high-risk (T1-4N0M0) prostate cancer patients that were treated within the Dutch seven-centre HYPRO trial between 2007 and 2010 [10,11,12]. In this trial, patients were randomized to HF (64.6 Gy in 19 fractions over 6.5 weeks) or CF (78 Gy in 39 fractions over 8 weeks). The target volume consisted of the prostate with or without seminal vesicles. The trial was approved on 5 April 2006 by the medical ethics committee of the Erasmus Medical Centre in Rotterdam, the Netherlands (06-045). The data of patients with at least 12 months of follow-up, up to 60 months (censoring data after clinical recurrence of disease, when applicable), with at least one follow-up visit after 12 months with toxicity evaluation were included in this study if 3D planning data were available. The data of one center were excluded because it only contained the dose data for one dose fractionation arm. Patient characteristics at the start of treatment and dose summary statistics are shown in Table S1.

### 2.2. Endpoint

The endpoint in this study was grade ≥ 2 late rectal bleeding (LRB) within 5 years post-treatment. We scored patients following the definitions provided by the Common Terminology Criteria for Adverse Events (CTCAE) and the Radiation Therapy Oncology Group and European Organization for Research and Treatment of Cancer (RTOG/EORTC). The CTCAE (v5) classifies rectal bleeding as a grade ≥ 2 event in the case of moderate to severe symptoms and/or if a treatment for bleeding is indicated [13]. The RTOG/EORTC consider intermittent bleeding as grade 2, late-radiation morbidity [14]. Therefore, we scored grade ≥ 2 bleeding if the case report indicated any form of medication or medical intervention for rectal bleeding such as hyperbaric oxygen treatment, transfusion or laser coagulation treatment. Additionally, we also scored LRB for patients who reported moderate bleeding on two or more follow-up questionnaires or severe bleeding on at least one questionnaire.

### 2.3. Dosiomic Features and Clinical Parameter

For BED calculations, both α/β = 2 Gy and α/β = 3 Gy were applied. An α/β ratio of 3 Gy is broadly assumed for late rectal toxicity [15,16,17,18]. We chose to perform the analysis for an α/β of 2 Gy as well, because 1.7 Gy (95% CI: 0.3–3.0) was found as the best fit value for grade ≥ 2 LRB specifically in a study that used CHHiP trial data [19].

**Figure 1:**
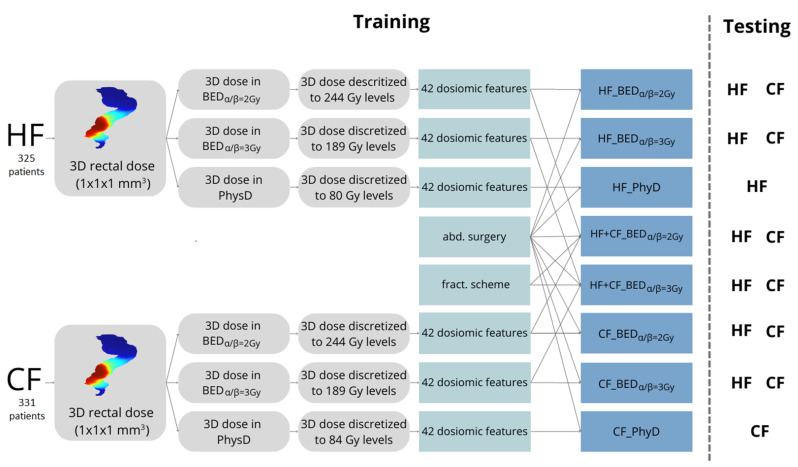
A flowchart of the study. The gray boxes indicate 3D dose distributions, the green boxes indicate features, and the blue boxes indicate models. The 3D rectal doses of HF and CF patients were extracted from the planned dose distributions and resampled to isotropic voxels. One dose distribution remained in a physical dose (PhyD) and two more were created by converting the dose to the BED with α/β = 2 Gy or 3 Gy. The dose distributions were then discretized to a number of levels corresponding to their range of doses. From the discretized PhyD, the BED_α/β=2Gy_ and BED_α/β=3Gy_ 3D doses, 42 dosiomic features were calculated. Subsequently, 8 models were developed, with 3 using HF data (HF_BED_α/β=2Gy_, HF_BED_α/β=3Gy_, HF_PhyD) and 3 using CF data (CF_BED_α/β=2Gy_, CF_BED_α/β=3Gy_, CF_PhyD). For these models, the 42 dosiomic features were candidate predictors in the model development (Section 2.4.4). Previous abdominal surgery was always included as a predictor in the final models. Further, 2 more models were developed using combined HF and CF data (HF+CF_BED_α/β=2Gy_, HF+CF_BED_α/β=3Gy_). For these models, the fractionation scheme was added as an extra candidate predictor. Lastly, HF_BED_α/β=2Gy_, HF_BED_α/β=3Gy_ CF_BED_α/β=2Gy_ and CF_BED_α/β=3Gy_ were tested on their training data and the data of the other fractionation scheme, HF_PhyD and CF_PhyD were only tested on their training data, and HF+CF_BED_α/β=2Gy_ and HF+CF_BED_α/β=3Gy_ were tested on their HF and CF training data separately.

Dosiomic feature calculation is described in detail elsewhere [3]. The study design including data collection and processing is visualized in Figure 1 and here, in short, is described. First, the 3D rectal dose distributions were obtained from the planned dose distributions. The rectum clinical contour was used as delineation from the ischial tuberosities to the bottom of the sacro-iliac joints including filling. Subsequently, the rectal dose distributions were resampled to isotropic 1 mm^3^ voxels based on B-spline interpolation. Second, one set of rectal dose distributions was kept in the physical dose (PhyD), and two more sets were created by converting the PhyD rectal dose distributions to the BED voxelwise by applying α/β = 2 Gy (BED_α/β=2Gy_) and 3 Gy (BED_α/β=3Gy_). Third, the three dose distributions were discretized to a number of levels that corresponded to their range of voxel doses (in Gy). PhyD distributions for HF and CF were discretized to 80 and 84 levels, respectively, corresponding to maximum voxel doses of 80 and 84 Gy. The BED_α/β=2Gy_ and BED_α/β=3Gy_ distributions were discretized to 244 and 189 levels, respectively, for both HF and CF, corresponding to maximum voxel doses of 244 Gy and 189 Gy in BED. Fourth, 42 dosiomics features were calculated from the rectal dose distributions in the PhyD, BED_α/β=2Gy_ and BED_α/β=3Gy_ sets. In each set, dosiomic features were subsequently normalized to range from 0 to 1 to reduce the feature magnitude impact. Dose levels will henceforth be referred to as gray levels, as is common in the texture analysis literature. The 42 dosiomic features were calculated from several matrices: 3 from the gray-level frequency histogram, 8 from the gray-level co-occurence matrix (GLCM), 13 from the gray-level run-length matrix (GLRLM), 13 from the gray-level size zone matrix (GLSZM) and 5 from the neighborhood gray-tone difference matrix (NGTDM) [20,21]. A list of all considered dosiomic features with descriptions is displayed in Table S2. Dosiomic features were obtained using MATLAB (The MathWorks Inc., Natick, MA, USA) based on Vallieres et al. [20].

Clinical parameters that have been found to be predictive for LRB in multiple studies were considered, which included ‘previous abdominal surgery’ and the ‘use of anticoagulation/cardiovascular history’ [22,23,24,25,26]. Since our dataset did not contain information on the use of anticoagulants or cardiovascular history, only previous abdominal surgery was included in the models.

### 2.4. NTCP Modelling

A total of 8 models were fitted (Figure 1, blue boxes) following the statistical procedure described below. The 42 dosiomic features were candidate predictors for these models. Previous abdominal surgery was always includedas a final predictor because of its established role in LRB. The 8 models were separated into three categories as follows.

#### 2.4.1. Part 1: Separate BED Models for HF and CF

Separate BED models were fitted using HF and CF data, as visible in Figure 1. HF_BED_α/β=2Gy_ and HF_BED_α/β=3Gy_ were trained using HF data and separately tested on the training data for the apparent performance and CF data for the test performance. Similarly, CF_BED_α/β=2Gy_ and CF_BED_α/β=3Gy_ were trained using CF data and separately tested on the training data for the apparent performance and HF data for the test performance (see Figure 1 for data usage).

#### 2.4.2. Part 2: Single Models for CF and HF Patients Together

BED models were also fitted to the combined HF and CF data together. These models, HF+CF_BED_α/β=2Gy_ and HF+CF_BED_α/β=3Gy_, were tested on the HF and CF training data separately (Figure 1). In addition to the 42 dosiomic features, the fractionation scheme was added as a candidate predictor for these models as follows: fractionation scheme = 1 and 0 for HF and CF patients, respectively.

#### 2.4.3. Part 3: Separate Physical Dose Models for HF and CF

Physical dose models were fitted to the HF (HF_PhyD) and CF (CF_PhyD) data separately. These models were only tested on their training data (Figure 1).

#### 2.4.4. Statistical Procedure

A 3-step statistical approach was followed to develop the models [3].

1. Feature selection

A total of 1000 bootstrap samples were drawn from the training data with replacement. For each bootstrap sample, the following steps were taken to select candidate predictors.

- The association between each candidate predictor and the outcome was determined with univariable logistic regression models. Candidate predictors with a *p*-value > 0.2 were rejected.
- Subsequently, multicollinearity was resolved by removing correlating candidate predictors. From the two predictors with the largest Spearman’s correlation coefficient between them, the one that was the least correlated to the outcome was removed. This was repeated until the variation inflation factor (VIF) ≤ 5 [26].
- The remaining predictors were used as candidate predictors in a multivariable logistic regression model with backwards elimination. The criterion for elimination was a change in deviance; if the *p*-value of the chi-squared test of the change in deviance after removing a predictor was larger than 0.01, the predictor was removed. The threshold of 0.01 was chosen to only allow the most predictive predictors to be selected. The remaining predictors in this bootstrap model made up the signature of the bootstrap sample.

2. Predictor coefficients

The final signature for the model consisted of abdominal surgery and the predictors that were selected in one-fourth or more of the bootstrap samples. If none fulfilled this requirement, the most selected predictor was added to the final signature. The choice of this threshold of one-fourth was chosen to allow a reasonable number of predictors to be selected. Multicollinear dosiomic features were removed from the final signature in the case of the VIF > 5.

A total of 1000 bootstrap resamples were drawn from the training data with replacement. Multivariable logistic regression models were fitted to each bootstrap sample with the predictors of the final signature. The final coefficients were obtained by taking the median of the 1000 coefficients. Given the final coefficients, the intercept for the final model was estimated by minimizing the negative log-likelihood. The odds-ratios (OR) for each predictor was calculated (as exponentiated by the coefficient) and reported to describe the effect size of the predictor in the model. The constant of the model was also calculated (as exponentiated by the intercept) and represented the baseline odds when all predictors equal 0.

3. Performance

The performance of the final model was evaluated with varying datasets (Figure 1). The model performances were evaluated by the area under the ROC curve (AUC) for discriminative power and the calibration slope and intercept as measures for calibration.

The models were developed and tested using R software (4.4.1, R Core Team, Vienna, Austria) [27] and RStudio (2024.04.2, Posit PBC, Boston, MA, USA) [28]. The toolboxes that were used were car, olsrr, dpyr, Tidyverse, predtools, pROC, glmtoolbox, MASS and rms [29,30,31,32,33,34,35,36,37].

## 3. Results

The 656 patients in the dataset consisted of 325 HF patients (56 events, 82 with previous abdominal surgery) and 331 CF patients (33 events, 91 with previous abdominal surgery).

### 3.1. NTCP Modelling

#### 3.1.1. Part 1: Separate BED Models for HF and CF

The separate HF BED and CF BED models (Table 1) and frequencies of feature selection (Figures S1 and S2) showed that which dosiomic feature is predictive for the outcome depends on the dose fractionation scheme. The final predictors in the HF BED and CF BED models included the LRHGE and LZHGE, which relate to high gray levels. HF_BED_α/β=2Gy_, however, only contained dosiomic features related to variance in the run-lengths and deviation from the mean gray level (RLV and VarianceGLCM, Table S2). For the HF BED models, VarianceGLCM and RLV were among the three most selected features in the bootstrap signatures (Figure S1), while they were barely selected for the CF BED models (Figure S2). The RLV and LZHGE were moderately correlated, with Spearman’s correlation coefficients of 0.65 and 0.70 for BED_α/β=2Gy_ and BED_α/β=3Gy_, respectively (Figure S3), whereas the VarianceGLCM and LZHGE were poorly correlated, with Spearman’s correlation coefficients of 0.12 and 0.13. This suggests that the RLV and LZHGE, to an extent, quantified the texture similarly, while the VarianceGLCM and LZHGE were independent. Univariate associations between the dosiomic features and the outcomes for the physical dose and BED are shown and discussed in the Supplementary Materials S2.

The HF BED models showed moderate discriminative ability with the training data, with AUCs of 0.68 and 0.69 (Table 2). The discriminative ability of HF_BED_α/β=2Gy_ was poor for CF, with an AUC of 0.55. HF_BED_α/β=3Gy_ showed a slightly better AUC of 0.62 for CF. The calibrations of these models for CF were poor, with calibration slopes and intercepts far from the ideal values of 1.00 and 0.00, respectively (Table 2). The negative calibration intercepts and calibration slopes of <1 indicated that the estimated probabilities for CF were generally too high and too extreme.

The CF BED models showed weak discriminative ability with the training data, with a AUCs of 0.62 and 0.63. These models showed AUCs of 0.65 for HF, which were better than the apparent performance (CF). The positive calibration intercepts and calibration slopes of >1 indicated that estimated probabilities for HF were generally too low and not extreme enough. Abdominal surgery only showed statistical significance (*p* > 0.05) in HF_BED_α/β=3Gy_ (Table 1).

#### 3.1.2. Part 2: Single Models for CF and HF Patients Together

**Figure 2:**
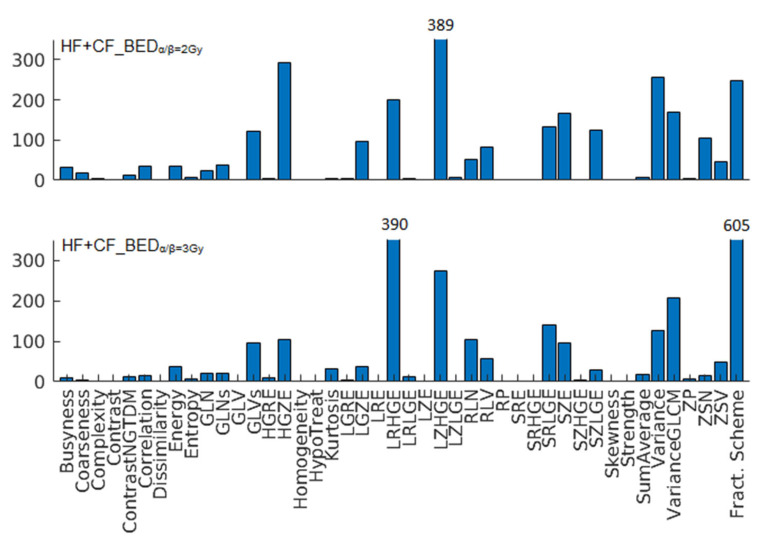
The frequency of dosiomic feature selection in the bootstrap signatures for the combined HF and CF models.

Both HF+CF_BED_α/β=2Gy_ and HF+CF_BED_α/β=3Gy_ contained dosiomic features related to high gray zones (Table 1). HF+CF_BED_α/β=2Gy_ also contained variance as a final predictor, while HF+CF_BED_α/β=3Gy_ contained the fractionation scheme. The HF+CF_BED models showed better discriminative ability with the CF data than the CF BED models, with AUCs of 0.64 and 0.69 compared to 0.62 and 0.63 (Table 2). In contrast, the HF+CF_BED models did not discriminate better with the HF compared to the HF BED models. Furthermore, in terms of calibration, the predictions were generally too high and extreme for HF and too low and not extreme enough for CF. The fractionation scheme was selected as a predictor in 247 and 605 bootstrap samples in the development of HF+CF_BED_α/β=2Gy_ and HF+CF_BED_α/β=3Gy_, respectively (Figure 2). Abdominal surgery showed statistical significance in both models (Table 1).

#### 3.1.3. Part 3: Separate Physical Dose Models for HF and CF

The physical dose models (HF_PhyD and CF_PhyD, Table 1) and frequencies of feature selection (Figures S1 and S2) showed that whether the dose was expressed as the BED or physical dose influenced which feature would be predictive for the outcome. HF_PhyD included Kurtosis as a predictor in the final model, even though this feature was of less importance during the development of the HF BED models (Figure S1). Similarly, RLN was the most selected feature in the bootstrap signatures for CF_PhyD, while it was of no importance in the CF BED bootstrap samples (Figure S2).

In terms of apparent performance, the physical dose models showed a discriminative ability in the training data that was similar to that of the BED models, with AUCs of 0.69 and 0.61 for HF and CF, respectively. Only in HF_PhyD, abdominal surgery showed statistical significance.

## 4. Discussion

Prostate RT with hypofractionated schedules has been broadly clinically adopted. As a broad range of schedules is used, there is a need for the development of toxicity prediction models that apply to all schedules. Since dosiomic features can add value to NTCP modelling [3,6], we evaluated such models for grade ≥ 2 LRB after CF or HF radiotherapy and investigated what the impact and added value was of applying the linear–quadratic model in the development of these models.

We found that the dose fractionation scheme partly determined which dosiomic feature was predictive for the outcome, that the BED models showed poor calibration with the data of the other fractionation scheme and that the physical dose models showed a similar apparent performance to that of the BED models. Furthermore, combining HF and CF only improved the discriminative ability for the CF data.

In both the HF BED and CF BED models, dosiomic features were selected that indicated a role for high doses in LRB. These findings are in line with the well-established predictive role of high-dose regions and the serial behavior of LRB [38]. In contrast to the CF BED models, in the HF BED models, deviation from the mean gray level was also predictive (Figures S1 and S2, Table S3).

Rossi et al. previously developed models for LRB for the physical dose using a set of HF patients from the HYPRO trial [3]. The candidate predictors in those models consisted of dosiomic features in combination with DVH and treatment-related parameters. The most selected features partly overlap with the ones in the physical-dose HF model in the current study. The difference could be due to an interaction between dosiomic features and the traditional dose parameter or treatment-related parameters, which were not considered in the current study.

To the best of our knowledge, the current study is the first to test the generalizability of dosiomics-based models to other fractionation schemes. The calibration slopes and intercepts of the HF BED models for CF and the CF BED models for HF were poor. However, models based on dosiomics relating to high gray values appeared to discriminate moderately with the data of the other fractionation, with AUCs of up to 0.65 (Table 2). Notably, the CF BED models showed worse AUCs in the training data (0.62 and 0.63) than in the HF data (0.65). The difference could be explained by the smaller number of events in CF (33) compared to HF (56).

Combining HF and CF data to obtain a larger dataset resulted in models with better discriminative ability with CF compared to the models fitted to just CF data (Table 2). With HF, however, these models did not perform better than the models fitted to just HF data. The fractionation scheme was selected much more frequently in the development of HF+CF_BED_α/β=3Gy_ than HF+CF_BED_α/β=2Gy_. This difference suggests that the dosiomic features explained the differences in outcomes between HF and CF better for α/β = 2 Gy than for α/β = 3 Gy. α/β = 2 Gy appears, therefore, to be more suitable to correct fractionation scheme differences than α/β = 3 Gy. This finding was also reported in a recent study in which the same dataset was used to develop LRB NTCP models with traditional dose (DVH) parameters [39]. Furthermore, it is in line with the 1.7 Gy (95% CI: 0.3–3.0) best-fit value for grade ≥ 2 LRB that Brand et al. found for Lyman–Kutcher–Burman models [19].

Whether doses were expressed as the BED or physical dose influenced which dosiomic features were selected (Figures S1 and S2). However, the apparent performances of the physical-dose models and the BED models were similar (Table 2). This result suggests that a dosiomics-based model for a single fractionation scheme can be developed with either the BED or physical dose. Puttanawurat et al. previously developed EQD2 and physical dosiomics-based NTCP models for radiation pneumonitis (RP) after esophageal cancer RT [8]. The majority of patients were treated with the same standard fraction size, and the EQD2 and physical models showed similar performance, which is in line with our findings.

In the current study, we found several impacts of applying the linear–quadratic model in dosiomics-based NTCP modelling for LRB that suggest that dosiomics-based modelling should be kept separate for different fractionations. However, in other studies, value was found in dosiomics-based modelling for multiple fractionation schemes. In a study by Zhou et al., predictive models for RP after non-small cell lung cancer were developed with data from various fractionation schemes [9]. In combination with radiomics, dosiomics from dose distributions in EQD2 were found to result in superior models compared those based on dosiomics from the dose distributions in physical dose. Furthermore, Avanzo et al. combined data from several fractionation schemes and found that BED dosiomics can predict radiation-induced fibrosis after partial breast irradiation [7].

## 5. Conclusions

In conclusion, the findings in the current study suggest that using the BED in the predictive dosiomic modelling of late rectal bleeding after prostate cancer radiotherapy to account for differences in fraction doses was of limited value. To achieve stable models, we propose that such models are developed in separate CF and HF datasets with either the BED or physical dose. For studies in which the data of multiple fractionation schedules are nonetheless combined for dosiomics-based modelling, we recommend also including an analysis addressing the schedules separately. We observed differences in which dosiomic features are predictive for HF and CF. Furthermore, the BED dosiomics models showed poor calibration with data of another fractionation scheme. Dosiomics models based on the BED performed similarly to models based on the physical dose in a single fractionation scheme. Only for CF, dosiomic models fitted to combined HF and CF data showed better discriminative ability than the models fitted to just CF data.

## Figures and Tables

**Table 1:** The final models with the median ORs and *p*-values of the predictors.

Model	Predictor	Median OR	Median *p*-Value
HF_BED_α/β=2 Gy_	VarianceGLCM	49.7	0.005
	RLV	4.4 × 10^−3^	0.004
	Abd. Surg.	1.87	0.058
	Constant (baseline odds)	0.10	
HF_BED_α/β=3 Gy_	LRHGE	71.2	<0.001
	Abd. Surg.	1.98	0.036
	Constant (baseline odds)	0.05	
CF_BED_α/β=2 Gy_	LZHGE	9.87	0.041
	Abd. Surg.	1.74	0.159
	Constant (baseline odds)	0.07	
CF_BED_α/β=3 Gy_	LZHGE	8.19	0.03
	Abd. Surg.	1.72	0.16
	Constant (baseline odds)	0.07	
HF+CF_BED_α/β=2 Gy_	Variance	17.88	0.020
	HGZE	3.27	0.202
	LZHGE	22.98	0.001
	Abd. Surg.	1.80	0.019
	Constant (baseline odds)	0.03	
HF+CF_BED_α/β=3 Gy_	LZHGE	2.72	0.319
	LRHGE	16.91	0.004
	Fr. Sch.	2.29	0.001
	Abd. Surg.	1.83	0.015
	Constant (baseline odds)	0.03	
HF_PhyD	Kurtosis	0.01	0.054
	LRHGE	288.9	<0.001
	Abd. Surg.	2.05	0.032
	Constant (baseline odds)	0.18	
CF_PhyD	RLN	0.31	0.272
	Abd. Surg.	1.70	0.172
	Constant (baseline odds)	0.11	

Abbreviations: OR = odds ratio; Abd. Surg. = abdominal surgery; Fr. Sch. = fractionation scheme; VarianceGLCM = variance gray-level co-occurrence matrix; RLV = run-length variance; LRHGE = long-run high gray-level emphasis; LZHGE = large-zone high gray-level emphasis; HGZE = high gray-level zone emphasis; RLN = run-length nonuniformity.

**Table 2:** Performances of final models with HF or CF data. Dark gray: patient set not used during model training (i.e., test data, test performance). Light gray: patient set used for model training (i.e., training data, apparent performance).

Model	Measure	HF Data	CF Data
HF_BED_α/β=2Gy_	AUC	0.68	0.55
	Cal Sl.	0.97	0.25
	Cal Int.	−0.04	−1.66
HF_BED_α/β=3Gy_	AUC	0.69	0.62
	Cal Sl.	0.98	0.64
	Cal Int.	−0.03	−1.29
CF_BED_α/β=2Gy_	AUC	0.65	0.62
	Cal Sl.	1.64	0.99
	Cal Int.	2.27	−0.02
CF_BED_α/β=3Gy_	AUC	0.65	0.63
	Cal Sl.	1.64	1.00
	Cal Int.	2.26	0.01
HF+CF_BED_α/β=2Gy_	AUC	0.66	0.64
	Cal Sl.	0.79	1.08
	Cal Int.	−0.59	0.28
HF+CF_BED_α/β=3Gy_	AUC	0.63	0.69
	Cal Sl.	0.75	1.28
	Cal Int.	−0.52	0.42
HF_PhyD	AUC	0.69	NA
	Cal Sl.	0.99	
	Cal Int.	−0.02	
CF_PhyD	AUC	NA	0.61
	Cal Sl.		0.99
	Cal Int.		−0.02

AUC = area under the curve; Cal Sl. = calibration slope; Cal Int. = calibration intercept; NA = not applicable.

## Data Availability

The data that support the findings of this study are available from the authors on request.

## Supplementary Materials

The following supporting information can be downloaded at https://www.mdpi.com/article/10.3390/cancers16244208/s1. Table S1: Patient characteristics at the start of treatment, dose summary statistics and incidence of LRB; Table S2: A list of the dosiomic features considered in this study; Table S3: Odds-ratios for dosiomic features with a *p*-value <0.1 in univariable analysis; Figure S1: The frequency of dosiomic feature selection in the bootstrap signatures for HF models; Figure S2: The frequency of dosiomic feature selection in the bootstrap signatures for CF models; Figure S3: Spearman’s correlations between dosiomic features in the final models calculated from BED dose distributions..
