# Supplementary material for: Added Value of Biological Effective Dose in Dosiomics-Based Modelling of Late Rectal Bleeding in Prostate Cancer
Source: Cancers (Basel). 2024 Dec 17;16(24):4208. doi: 10.3390/cancers16244208 (PMC11674648; doi:10.3390/cancers16244208)
Supplement: Supplementary file 1 [file cancers-16-04208-s001.zip › cancers-3286958-supplementary.pdf]

Supplementary Materials

Section S1: Study population characteristics

Table S1. Patient characteristics at the start of treatment, dose summary statistics and incidence of LRB.

Variable	HF (N=325)	CF (N=331)
Patient characteristics		
Age, mean (standard deviation) in years	69.5 (6.5)	70.4 (5.8)
TURP, n (%)	28 (8)	35 (11)
Previous abdominal surgery, n (%)	82 (25)	91 (27)
Diabetes, n (%)	47 (14)	42 (13)
Adjuvant hormonal therapy, n (%)	210 (65)	220 (66)
Dose summary statistics		
Treatment schedule	19 x 3.4 Gy	39 x 2 Gy
Mean rectal dose, median (range) in Gy	30.8 (12.6 – 48.4)	36.8 (12.0 – 55.0)
D_{1cm3}, median (range) in Gy	63.7 (59.4 – 67.1)	76.2 (70.9 – 81.5)
Outcome		
Grade ≥2 Late rectal bleeding, n (%)	56 (17)	33 (10)

Abbreviation: HF = Hypofractionation, CF = Conventional Fractionation, TURP = transurethral resection of prostate, D_{1cm3} = minimum dose in the most exposed 1 cm^3

Section S2: Dosiomic features

Table S2. A list of the dosiomic features considered in this study [20]. Copied from Rossi et al [3].

**Frequency histogram**	
Variance	Measure of grey level frequency homogeneity
Skewness	Measure of histogram asymmetry
Kurtosis	Measure of histogram ”peakedness”
**Grey level Co-Occurrence Matrix**	
Energy	Measure of local uniformity of grey levels
Contrast	Measure of the amount of grey levels
Entropy	Measure of randomness of grey level
Homogeneity	Measure of local homogeneity, higher with less contrast
Correlation	Measure of neighbouring pixel grey level linear dependency
Sum Average	Measure of overall image brightness
Dissimilarity	Measure of spread out of grey levels sum of pixel pair
VarianceGLCM	Measure of deviation from average values
**Grey level Run Length Matrix**	
SRE	Short Run Emphasis
LRE	Long Run Emphasis
GLN	Grey Level Nonuniformity
RLN	Run Length Nonuniformity
RP	Run Percentage

LGRE	Low Grey Level Run Emphasis
HGRE	High Grey Level Run Emphasis
SRLGE	Short Run Low Grey Level Emphasis
SRHGE	Short Run High Grey Level Emphasis
LRLGE	Long Run Low Grey Level Emphasis
LRHGE	Long Run High Grey Level Emphasis
GLV	Grey Level Variance
RLV	Run Length Variance
**Grey Level Size Zone Matrix**	
SZE	Small Zone Emphasis
LZE	Large Zone Emphasis
GLNs	Grey Level Nonuniformity (second one, as for GLRLM)
ZSN	Zone Size Nonuniformity
ZP	Zone Percentage
LGZE	Low Grey Level Zone Emphasis
HGZE	High Grey Level Zone Emphasis
SZLGE	Small Zone Low Grey Level Emphasis
SZHGE	Small Zone High Grey Level Emphasis
LZLGE	Large Zone Low Grey Level Emphasis
LZHGE	Large Zone High Grey Level Emphasis
GLVs	Grey Level Variance (second one, as for GLRLM)
ZSV	Zone Size Variance
**Neighborhood Grey Tone Difference Matrix**	
Coarseness	Measure of image granularity
Contrast	Measure of fluctuation of tone levels
Busyness	Measure of rate of tone changes
Complexity	Measure of sum of normalized differences between tones
Strength	Integration of busyness and complexity

Section S3: Univariate associations

Table S3. Odds-ratios for dosiomic features with a p-value <0.1 in univariable analysis. The Odds-Ratios of significant associations (p-value < 0.05) are indicated with a asterisk (*).

	**HF in BED (α/β=2Gy)**	**HF in BED (α/β=3Gy)**	**HF in physical dose**	**CF in BED (α/β=2Gy)**	**CF in BED (α/β=3Gy)**	**CF in physical dose**	**HF+CF in BED (α/β=2Gy)**	**HF+CF in BED (α/β=3Gy)**
**Frequency histogram**								
Variance	27.7*	22.2*						
Kurtosis	0.04	0.03*	0.006*					
**Grey level Co-Occurrence Matrix**								
Contrast			0.10					
Correlation	21.4*	22.3*						
Dissimilarity			0.15					
VarianceGLCM	28.6*	22.7*	9.71					
**Grey level Run Length Matrix**								
SRE			0.15*					
RLN			0.15*					
HGRE	3.83*							
LRHGE	31.1*	63.0*	285.5*	10.4*	11.9*	17.8*	10.4*	11.9*
RLV	0.01*	0.02*	0.13*					
**Grey Level Size Zone Matrix**								
SZE	0.18	0.22			0.19			0.19
ZSN	0.20				0.15			0.15
ZP	0.22	0.19	0.02					
LZHGE	475*	117*	12.4*	12.0*	10.0*	34.7*	12.0*	10.0*
GLNs	12.6	8.7						
GLVs	0.01	0.005	0.01					
**Neighborhood Grey Tone Difference Matrix**								
Coarseness			0.22					

Among the univariately significantly associated features in HF BED data were dosiomics related to grey level non-homogeneity (Table S2). These features, VarianceGLCM, Variance, RLV and Kurtosis, were not associated with the outcome in CF data. The two features with a significant association with the LRB in CF BED data (LRHGE and LZHGE) however, also showed association in HF BED data.

Section S4: NTCP models

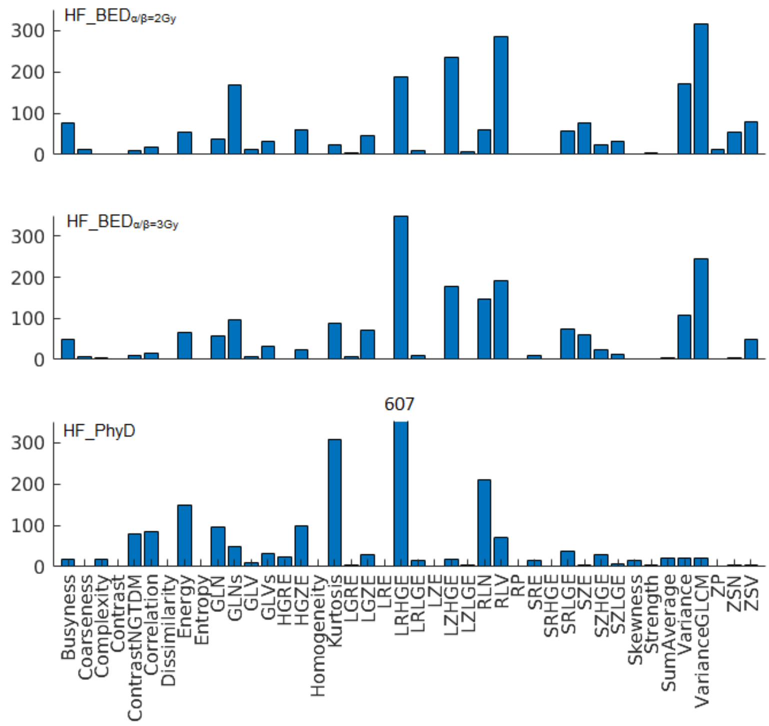

Figure S1. The frequency of dosiomic feature selection in the bootstrap signatures for HF models

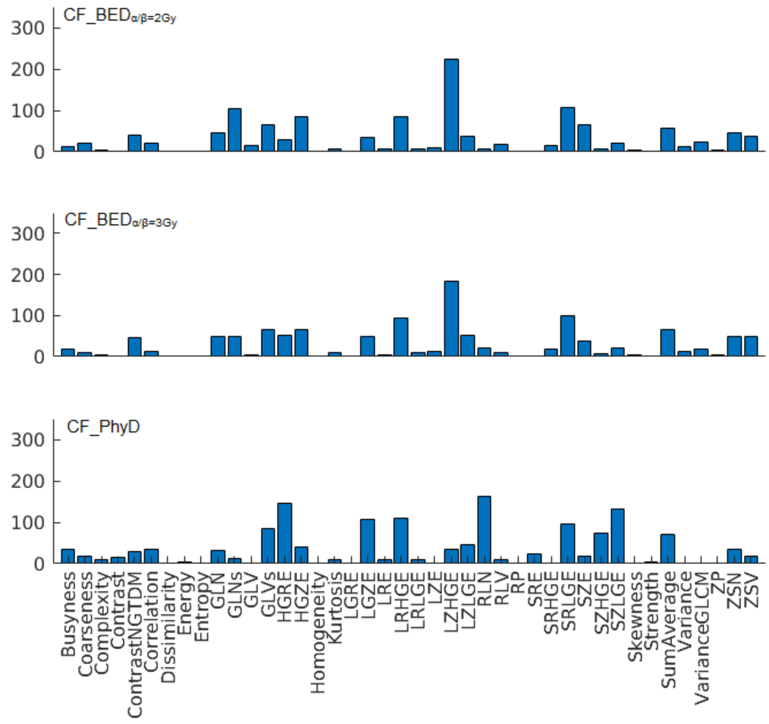

Figure S2. The frequency of dosiomic feature selection in the bootstrap signatures for CF models

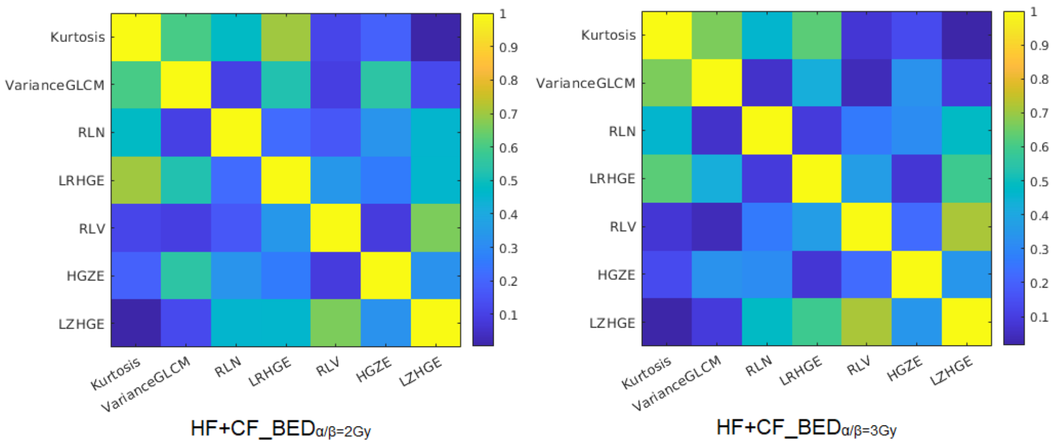

Figure S3. Spearman correlations between dosiomic features in the final models calculated from BED dose distributions.
